# Counteracting the activation of pAkt by inhibition of MEK/Erk inhibition reduces actin disruption-mediated apoptosis in PTEN-null PC3M prostate cancer cell lines

**DOI:** 10.3892/ol.2013.1547

**Published:** 2013-08-26

**Authors:** YONG-TAE AHN, IK JAE SHIN, JONG-MYOUNG KIM, YOUN SOOK KIM, CHU LEE, SEONG-A. JU, WON G. AN

**Affiliations:** 1 Department of Biomedical Science, Kyungdong University, Goseong-gun, Gangwon-do 219-832, Republic of Korea; 2 School of Korean Medicine, Pusan National University, Yangsan, Busan 626-870, Republic of Korea; 3 Department of Marine Biomaterials and Aquaculture, Pukyong National University, Busan 608-737, Republic of Korea; 4 School of Medicine, Pusan National University, Yangsan, Busan 626-870, Republic of Korea; 5 Aquaculture Industry Division, National Fisheries Research and Development Institute, Gangneung, Gangwon-do 210-861, Republic of Korea; 6 Biomedical Research Center, Ulsan University Hospital, College of Medicine, University of Ulsan, Ulsan 682-714, Republic of Korea

**Keywords:** latrunculin B, PTEN-null PC3M prostate cancer cell lines, apoptosis, pAkt, MEK/Erk, actin

## Abstract

The actin cytoskeleton is important in the maintenance of cellular homeostasis and in signal transduction pathways leading to cell growth and apoptotic cell death in eukaryotic cells. Disruption of actin dynamics is associated with morphological changes in cancer cells. Deletion of phosphatase and tensin homolog (PTEN), a tumor suppressor gene involved in the regulation of the cell cycle and apoptosis, leads to cytoskeleton disruption and double-strand breaks (DSBs). To study the mechanism(s) of actin disruption-mediated apoptosis and its potential application for anticancer therapy, PTEN-null PC3M prostate cancer cells were treated with latrunculin B (LB). LB induced destabilization of the actin microfilament and apoptosis in a dose-dependent manner, as demonstrated by morphological changes and nuclear condensation in the PC3M cells. In addition, it resulted in an increase in the levels of γH2AX recruitment, implicating the induction of DNA damage, including DSBs. Induction of Bax, with little effect on Bcl-2 expression, indicated that actin disruption causes apoptosis through activation of Bax signaling in PC3M cells. Treatment with U20126, a mitogen-activated protein kinase kinase (MEK) inhibitor, resulted in attenuated induction of DSBs and apoptosis through activation of protein kinase B (Akt), suggesting that LB-mediated actin dysfunction induces DSBs via the MEK/extracellular signal-regulated kinase (Erk) pathway in cells. Therefore, counteracting activation of phosphorylated Akt stemming from the inhibition of MEK/Erk resulted in attenuation of actin disruption-induced apoptotic events in the PC3M cells. The results of this study provide information not only for use in delineation of the molecular association between actin disruption and tumorigenesis, but also for the development of a strategy for actin-based anticancer chemotherapy against highly metastatic prostate cancer.

## Introduction

Extracellular signal-regulated kinase (Erk), a member of the mitogen-activated protein kinase (MAPK) family, regulates cellular responses to signals that lead to cell growth, proliferation and differentiation, as well as DNA damage (1,2). Dysfunction of phosphatase and tensin homologue (PTEN) is one of the main causes of cancer progression and metastasis (3,4). Deletion of PTEN has been demonstrated to result in elevation of phosphorylated protein kinase B (pAKT) levels, which in turn resulted in suppression of Erk signaling, in prostate cancer (5). Therefore, Ras/Erk signaling may be an effective target for the treatment of advanced metastatic prostate cancer cells. Erk signaling is associated with cancer cell proliferation and DNA damage; however, the detailed mechanism of Ras/Erk signaling in cancer cells remains unclear (6).

Actin cytoskeleton, the most abundant and ubiquitous protein, is important in various signaling pathways involved in cellular migration, adhesion, metastasis, cytokinesis, chromatin remodeling and transcriptional processes. Malignant cancer cells have distinct features, including modified cellular morphology and migration machinery, and disrupted cell division. As the changes are associated with actin dynamics, understanding the mechanism of actin disruption in cancer cells is important for the development of cancer chemotherapy (7). Small molecules targeting actin microfilaments have been tested for modulation of the rapid growth and proliferation of cancer cells (8). The small molecule latrunculin B (LB) inhibits actin polymerization and nucleotide exchange in G-actin by binding to the monomer in a one to one complex, without affecting the organization of the microtubular system (9,10).

PTEN interacts with actin cytoskeleton remodeling at focal adhesions of the cell membrane and inhibits phosphatidylinositide 3-kinase (PI3K) (11,12). In addition, it regulates the repair of impaired DNA double-strand breaks (DSBs) and nucleotide excision repair (13,14). Thus, it was hypothesized that disruption of actin may be a potential therapy for the treatment of highly metastatic prostate cancer. The current study investigated the effect of disruption of actin microfilaments on DSBs, the cell cycle and apoptotic signaling with LB treatment, to elucidate the mechanism of actin disruption in PTEN-null PC3M prostate cancer cells.

## Materials and methods

### Chemicals and antibodies

The metastatic human prostate cancer PC3M cell line was obtained from the American Type Culture Collection (Manassas, VA, USA). The cells were incubated in Dulbecco’s modified Eagle’s medium supplemented with 1% antibiotic-antimycotic solution and 10% fetal bovine serum (Gibco-BRL, Carlsbad, CA, USA) at 37°C in a humidified atmosphere of 5% CO_2_. To identify the cell signals activated upon treatment with/without LB, LY294002, U0126 or N-acetyl L cysteine (NAC), experiments were performed in the absence of serum and exogenous growth factors. LB was purchased from Calbiochem-Novabiochem Corp. (San Diego, CA, USA); while LY294002, U0126, NAC, RNase A and propodium iodide (PI) were purchased from Sigma Aldrich (St Louis, MO, USA). Rhodamine-phalloidin and Molecular Probes^®^ were obtained from Invitrogen Life Technologies (Carlsbad, CA, USA), and Complete Protease Inhibitor Cocktail tablets were purchased from Roche (Mannheim, Germany). Protran^™^ nitrocellulose membranes were purchased from Whatman International Ltd. (Maidstone, UK) and a BCA Protein Assay kit was obtained from Pierce (Rockford, IL, USA). Enhanced chemiluminescence (ECL) Western Blotting Detection Reagents, and sheep anti-mouse monoclonal IgG (HRP-linked) and donkey anti-rabbit polyclonal IgG (HRP-linked) antibodies were purchased from GE Healthcare (Piscataway, NJ, USA); while anti-rabbit poly (ADP-ribose) polymerase (PARP), pAkt and phosphorylated Erk (pERK) polyclonal antibodies were purchased from Cell Signaling Technology, Inc. (Danvers, MA, USA). Anti-Bax and anti-Bcl-2 antibodies were purchased from Santa Cruz Biotechnology, Inc. (Santa Cruz, CA, USA) and anti-phospho-histone H2A X antibody (Ser139; clone, JBW301) was obtained from Upstate Biotechnology, Inc. (Lake Placid, NY, USA).

### Viability assay

Cells (2×10^4^/well) were incubated in 96-well flat bottom plates in 100 μl culture medium (Dulbecco’s modified Eagle’s medium supplemented with 1% antibiotic-antimycotic solution and 10% fetal bovine serum) and stabilized in a humidified atmosphere of 5% CO_2_ at 37°C for >12 h. The cells were exposed to LB (50, 75, 100, 150, 200 and 300 nM) for 24 h. The MTT assay (Abnova, Taipei City, Taiwan) was performed according to the manufacturer’s instructions. Briefly, 20 μl 5 μg/ml MTT was added to each well and incubated for 4 h at 37°C. Then, the medium was carefully removed. DMSO was added to the reactants and mixed gently on an orbital shaker for 1 h at room temperature. The absorbance at 570 nm was determined using a Victor^3™^ multilabel plate reader (model 1420; Perkin-Elmer, Waltham, MA, USA). Triplicate wells were assayed for each condition.

### Immunocytochemistry

Immunocytochemistry was performed according to the manufacturer’s instructions (Invitrogen Life Technologies) with certain modifications. The cells growing on the cover slip were washed with cold phosphate-buffered saline (PBS). For permeabilization, cells were incubated with 150 μl permeabilizing buffer (PB; 0.15 M NaCl, 10 mM Tris, 1 mM MgCl_2_, 0.2 mM dithiothreitol, 0.5 mM CaCl_2_ and 25% glycerol; pH 8.0) for 4–5 min, 150 μl PB with 0.5% Triton X-100 for 3.5 min, 150 μl PB for 30 min at 37°C and then washed with PBS with 0.2% Tween-20 (PBS-T). Cells were fixed in 4% paraformaldehyde (pH 7.2) for 15 min and washed twice with PBS-T. Before cell staining, cells were blocked in PBS-T with 10% FBS for 20 min. Rhodamine-phalloidin (1:200) and DAPI (0.5 mg/ml) were applied for 60 min in the dark for staining of F-actin and DNA, respectively. Following washing with PBS-T, phase contrast and fluorescent images of the cells were observed using a confocal microscope (Olympus IX81; Olympus, Tokyo, Japan).

### Cell extraction

Harvested cells were incubated in a lysis buffer consisting of one Complete Protease Inhibitor Cocktail tablet (Roche) to 50 ml TNES buffer (50 mM Tris-HCl, pH 7.4; 1% nonidet P-40; 2 mM EDTA; 100 mM NaCl) on ice for 30 min. Following centrifugation at 13,000 × g for 10 min, the supernatant was stored at −80°C until use. Nuclear extracts were prepared according to the protocol of the Clontech Transfactor Extraction kit (Clontech Laboratories Inc., Mountain View, CA, USA). Cells were centrifuged at 450 × g for 5 min, washed in PBS, then 5X volume of ice-cold Buffer A [50 mM Tris-HCl (pH 7.4), 1.5 mM MgCl_2_, 10 mm KCl, 1 mM dithiothreitol, 1 mM phenylmethanesulfonylfluoride (PMSF) and protease inhibitors] were added to the cell pellet. After 15 min incubation on ice, the cells were centrifuged at 420 × g at 4°C for 5 min and resuspended in 2X volume of Buffer A. The mixture was homogenized with ten strokes of a glass dounce homogenizer and then centrifuged at 11,000 × g for 20 min. The resulting pellet consisting of the isolated nuclei was further homogenized in two to three volumes of 50 mM Tris-HCl (pH 7.4), 0.42 mM NaCl, 1.5 mM MgCl_2_, 0.2 mM EDTA, 25% glycerol and 1 mM PMSF. The nuclear suspension was rotated gently for 30 min at 4°C followed by centrifugation at 13,000 × g for 30 min. The amount of protein was determined using the Bradford protein assay (Bio-Rad Laboratories, Inc., Hercules, CA, USA).

### Western blot analysis

Total cellular extracts (30 μg) and nuclear extracts (5 μg) were resolved by 8 and 12% sodium dodecyl sulfate-polyacrylamide gel electrophoresis, respectively, and transferred onto a nitrocellulose membrane by electroblotting. The membranes were soaked in TBS-T buffer [20 mM Tris, pH 7.4; 137 mM NaCl; 0.1% (v/v) Tween-20] containing 5% (w/v) non-fat milk, and were probed with the primary antibodies diluted in TBS-T [PARP (1:1000), cleaved PARP (1:10000), Bcl-2 (1:1000), Bax (1:500), tubulin (1:500), pAkt (1:1000), pErk (1:1000) and γH2AX (1:8000)]. Detection was performed using the horseradish peroxidase-linked secondary antibody, followed by ECL. Anti-tubulin antibody (Santa Cruz Biotechnology, Inc.) was applied to confirm equal loading of the proteins and successful transfer to the membranes.

### Flow cytometry analysis of the cell cycle

The cells were treated with 150 nM LB and/or 20 μM U0126 in 6-well plates in a humidified atmosphere of 5% CO_2_ at 37°C for 6–24 h. The cells were washed with ice-cold PBS, fixed in 100% ethanol, and maintained at −20°C for up to two weeks before analysis of DNA content. Cell cycle analysis was performed using cells stained with PI solution, containing 50 g/ml PI, 0.1% nonidet P-40, 0.1% sodium citrate and 100 g/ml RNase A, in the dark at 37°C for 30 min.

### Statistical analysis

Data are presented as the mean ± standard error of at least three independent experiments, and images shown are representative of three to six independent experiments.

## Results

### LB induces cell growth inhibition in PTEN-null PC3M cells

**Figure 1:**
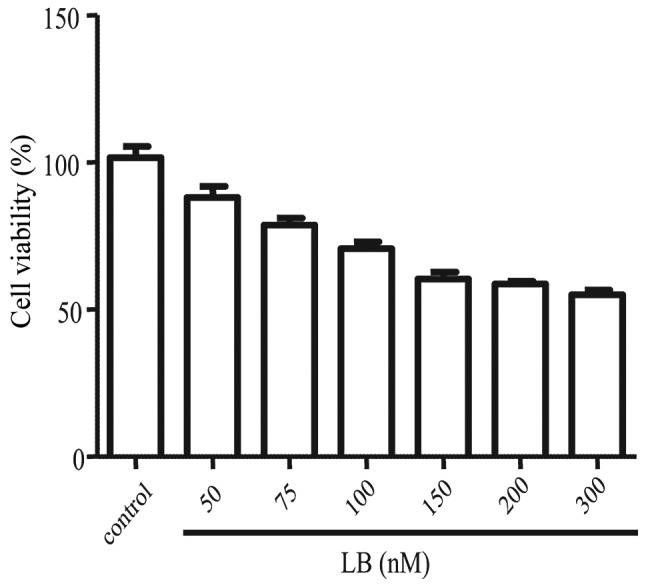
Effect of LB on the viability of PTEN-null PC3M prostate cancer cells. PTEN-null PC3M cells were treated with different concentrations of LB for 24 h, and then cell viability was determined by MTT assay. The values are represented as the percentage cell viability, where untreated control cells were regarded as 100% (mean ± SE, n=3). LB, latrunculin B; PTEN, phosphatase and tensin homolog.

The cytotoxicity of an actin-disrupting agent is an important encumbrance in the development of anticancer drugs based on an actin target. Preliminary analysis of cell proliferation under different concentrations of LB for 24 h indicated that an IC_50_ value of LB was 417 nM in the PC3M cells (data not shown). To determine an appropriate concentration of LB for the cancer cells, the MTT assay was performed (Fig. 1). The viability of the cells decreased in a dose-dependent manner upon treatment with various concentrations of LB (50–300 nM) for 24 h. The cell viability particularly decreased (to below ~65%), compared with that of the control, with LB treatment at concentrations higher than 150 nM.

### LB destabilizes actin microfilaments and induces apoptosis via enhancement of the expression of Bax in PTEN-null PC3M cells

**Figure 2:**
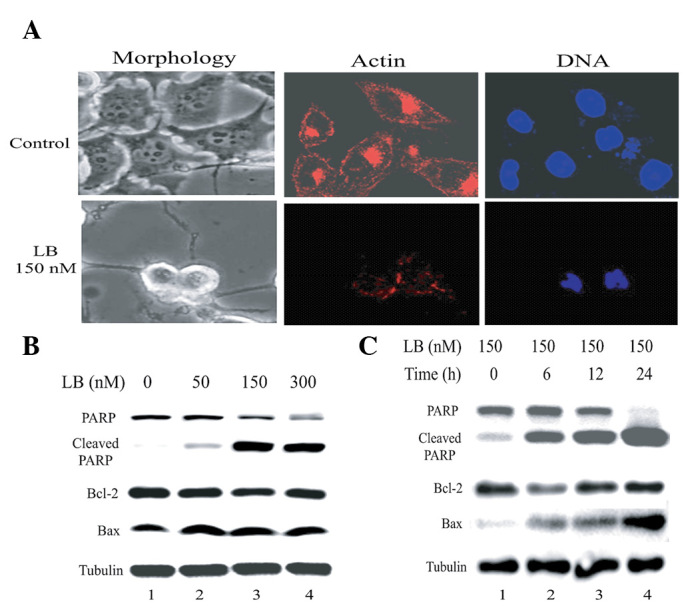
LB-mediated actin disruption induces apoptosis via enhancement of Bax expression in PTEN-null PC3M prostate cancer cell lines. (A) Disruption of actin microfilament and induction of apoptosis. The morphology, immunostained F-actin (rhodamine-phalloidine) and DNA (DAPI) of PC3M cells, with or without treatment (150 nM LB for 24 h), were examined by phase contrast microscopy and confocal microscopy. (B and C) Cleaved PARP, as well as Bcl-2 and Bax proteins, were expressed following LB treatment. PC3M cells were treated with either different concentrations (0, 50, 150 and 300 nM) of LB for 24 h (B) or with different times (0, 6, 12 and 24 h) under fixed 150 nM LB (C). Total cell extracts (30 μg) were used for western blotting with anti-PARP, -Bcl-2 and -Bax antibodies. LB, latrunculin B; PTEN, phosphatase and tensin homolog; PARP, poly (ADP-ribose) polymerase.

To study the molecular mechanism of actin disruption-mediated apoptosis in prostate cancer cells, PTEN-null PC3M cells were treated with 150 nM LB (Fig. 1). Morphological analysis was performed on the PC3M cells incubated with 150 nM LB for 24 h using phase contrast microscopy (Fig. 2A). The cells became rounded in shape with characteristics that included cytoplasmic shrinkage and marked convolution of the cellular surface whilst remaining attached to the plate. A well-developed actin cytoskeleton with numerous subcortical actin filaments and stress fibers was observed in the control PC3M cells; however, F-actin and irregular aggregates of phalloidin-reactive materials were observed in cells treated with LB (Fig. 2A). Staining of the PC3M cells with DAPI and rhodamine-phalloidin, followed by confocal microscopy analysis, was also performed for evaluation of apoptosis. Nuclear condensation and apoptotic bodies, hallmarks of apoptosis, were observed in cells treated with LB (Fig. 2A). Consequently, the results indicated that a disruption of the actin microfilament and induction of apoptosis occurred in the cells upon treatment with LB.

Induction of apoptosis was further confirmed by the levels of proteins that are involved in apoptotic signal transduction pathways, using western blot analysis. This was performed using the cells incubated with different concentrations (0, 50, 150 and 300 nM) of LB for 24 h (Fig. 2B). The PC3M cells without LB treatment showed expression of an uncleaved 116-kDa PARP protein (Fig. 2B; lane 1), whilst the cells treated with different concentrations of LB showed a progressive decrease in expression of the 116-kDa PARP (Fig. 2B; lanes 2 to 4). Additionally, under the same conditions, expression of the cleaved 89-kDa PARP fragment showed a marked increase from 0–50 nM LB and from 50–150 nM LB, but not from 150–300 nM LB. No difference was observed in the expression levels of Bcl-2 and Bax under the conditions (Fig. 2B). Subsequently, to identify an optimal time condition, the experiment was carried out using different time periods (0, 6, 12 and 24 h) with 150 nM LB (Fig. 2C). The PC3M cells without incubation time showed expression of uncleaved 116-kDa PARP protein (Fig. 2C; lane 1), which decreased with incubation time (Fig. 2C; lanes 2 to 4). Moreover, expression of the cleaved 89-kDa PARP fragment increased under the same conditions. The level of Bax expression, but not of Bcl-2, increased in the cells as the incubation time increased.

Therefore, the results supported the theory that actin disruption causes apoptosis through activation of both PARP and Bax signaling in PC3M cells.

### LB induces DNA DSBs via the mitogen-activated protein kinase kinase (MEK)/Erk pathway

**Figure 3:**
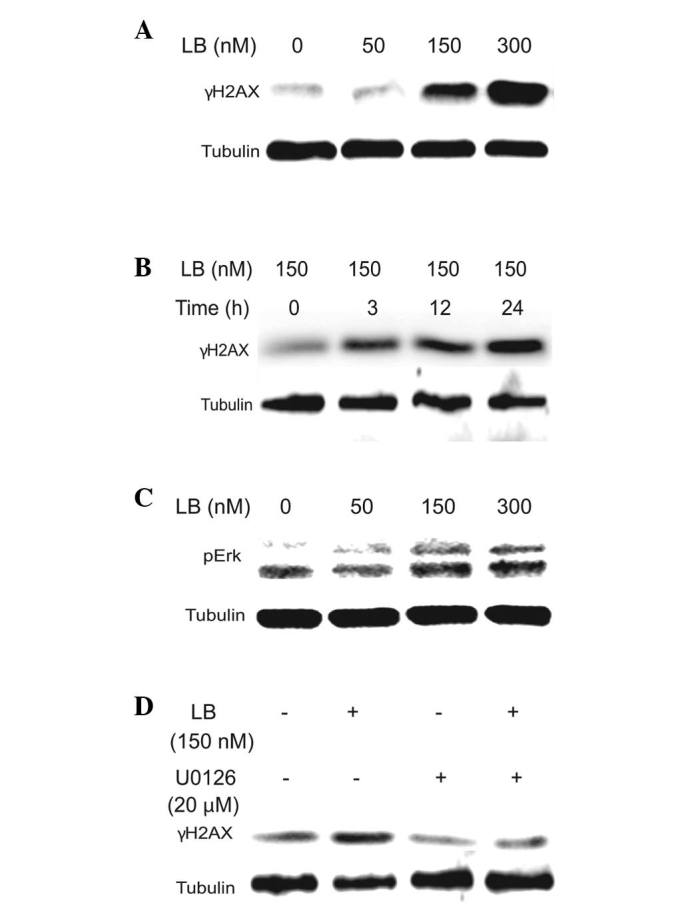
LB induces DSBs via the MEK/Erk pathway in PTEN-null prostate cancer cells. (A and B) Levels of γH2AX protein expressed following LB treatment. PC3M cells were treated with either LB (0, 50, 150 and 300 nM) for 24 h (A) or with 150 nM LB for 0, 3, 12 and 24 h (B). (C) Levels of pErk protein induced after LB treatment. PC3M cells were treated with LB (0, 50, 150 and 300 nM) for 24h. (D) Effect of LB treatment, with or without U0126, on γH2AX expression. PC3M cells were treated with LB (150 nM) in the presence or absence of U0126 (20 μM) for 24 h. Nuclear extracts (5 μg) for γH2AX and total cell extracts (30 μg) for pErk were used for western blotting with anti-γH2AX and anti-pErk antibodies, respectively. LB, latrunculin B; DSBs, double-strand breaks; MEK, mitogen-activated protein kinase kinase; Erk, extracellular signal-regulated kinase; PTEN, phosphatase and tensin homolog; pErk, phosphorylated Erk.

The level of γH2AX protein expression in the PC3M cells was measured to elucidate the effect of actin destabilization on genome integrity (Fig. 3). Treatment with LB resulted in an increase in the levels of γH2AX proteins recruited to the site of DNA damage in an LB dose- and time-dependent manner (Fig. 3A and B). As PC3M cells have a deleted PTEN tumor suppressor gene, LB-induced DNA damage analysis focused on Erk, one of the effectors responding to DNA damage. Activation of Erk signaling, which reflects LB-induced DSBs, was measured by the expression levels of pErk in the PC3M cells treated with 0, 50, 150 and 300 nM LB for 24 h. The level of pErk expression showed a gradual increase as the LB concentration increased (Fig. 3C). To assess whether inhibition of MEK/Erk signaling attenuates the production of DSBs, the levels of γH2AX expression were analyzed in cells treated with 150 nM LB for 12 h in the presence or absence of 20 μM U0126, an MEK/ERK inhibitor. Inhibition of ERK signaling resulted in abrogation of γH2AX protein expression activated by LB (Fig. 3D).

Therefore, the results supported the theory that actin disruption induced DSB-triggered apoptosis via the MEK/Erk signaling pathway in PC3M cell lines.

### Inhibition of MEK/Erk signaling attenuates LB-mediated apoptosis in PC3M cell lines

**Figure 4:**
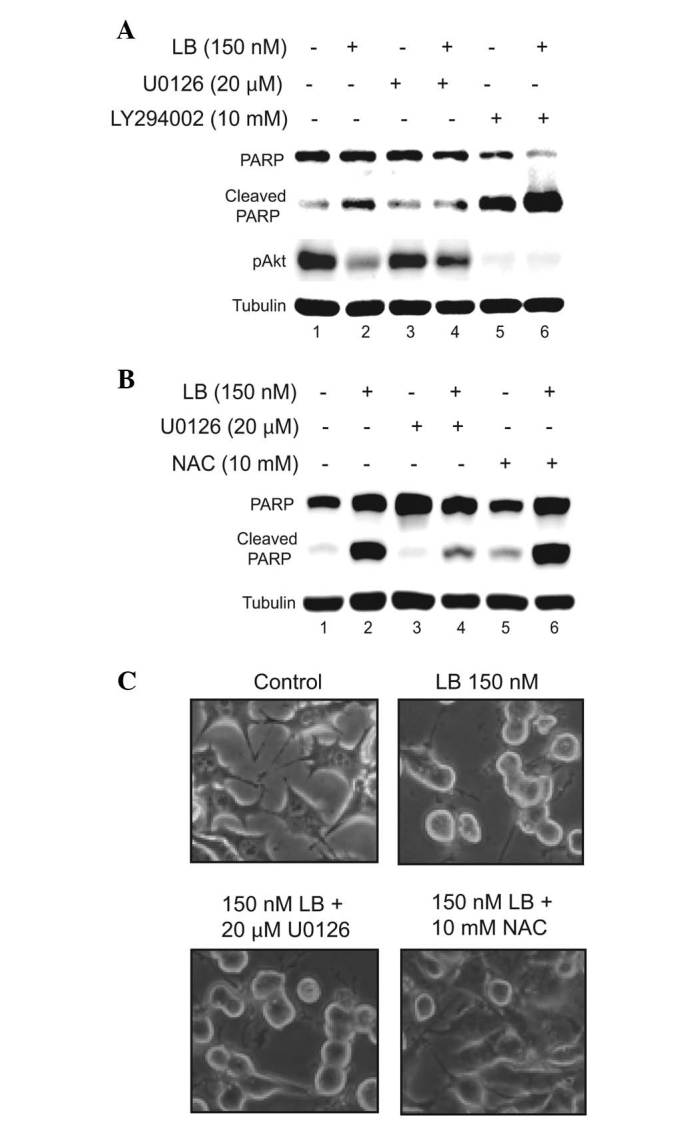
U0126 attenuates LB-mediated apoptotic events in PTEN-null PC3M cells. (A) Levels of PARP cleavage and pAkt after treatment with LB in the presence or absence of U0126 and LY294002. PC3M cells were treated with LB (150 nM) in the presence or absence of U0126 (20 μM) and LY294002 (10 μM), respectively, for 24 h. (B and C) Reduction of LB-mediated morphological change and PARP cleavage by Erk inhibition, but not by ROS inhibition. PC3M cells were treated with LB (150 nM) in the presence or absence of U0126 (20 μM) and NAC (10 mM), respectively, for 24 h. Total cell extracts (30 μg) were used for western blotting with anti-PARP and anti-pAkt antibodies, respectively. LB, latrunculin B; PTEN, phosphatase and tensin homolog; PARP, poly (ADP-ribose) polymerase; pAKT, phosphorylated protein kinase B; Erk, extracellular signal-regulated kinase; ROS, reactive oxygen species; NAC, N-acetyl-L-cysteine.

To determine whether inhibition of the Ras/MEK/Erk or the PI3K/PTEN/Akt pathway affects LB-mediated apoptosis, the PC3M cells treated with 150 nM LB for 24 h were further incubated with 20 μM U0126 or 10 μM LY294002, a PI3K inhibitor. Suppression of pAkt expression indicated that LB may induce apoptosis in PC3M cell lines (Fig. 4A; lanes 5 and 6). Inhibition of MEK/Erk by U0126, but not inhibition of PI3K signaling by LY294002 was observed, implying that pAkt activity confers resistance to LB-mediated apoptosis (Fig. 4A, lanes 3 and 4). To determine whether LB-mediated apoptosis is involved in the production of reactive oxygen species (ROS) and the activation of Erk, cells treated with 150 nM LB for 24 h were further incubated with 10 mM NAC (Fig. 4B and C). Typical apoptotic morphological changes, including shrinkage of the cytoplasm, membrane blebbing and condensation of nuclei, were observed in PC3M cells treated with LB, with or without NAC. However, marked suppression of LB-induced apoptotic features was observed in cells treated with a combination of LB and U0126 (Fig. 4C).

To confirm the results of the morphological analysis (Fig. 4C), the level of PARP cleavage was also investigated (Fig. 4A and B). As expected, co-treatment with U0126 resulted in suppression of LB-induced PARP cleavage (Fig. 4A and B; lanes 2 and 4), whereas NAC did not affect LB-mediated apoptosis (Fig. 4B; lanes 5 and 6).

**Figure 5:**
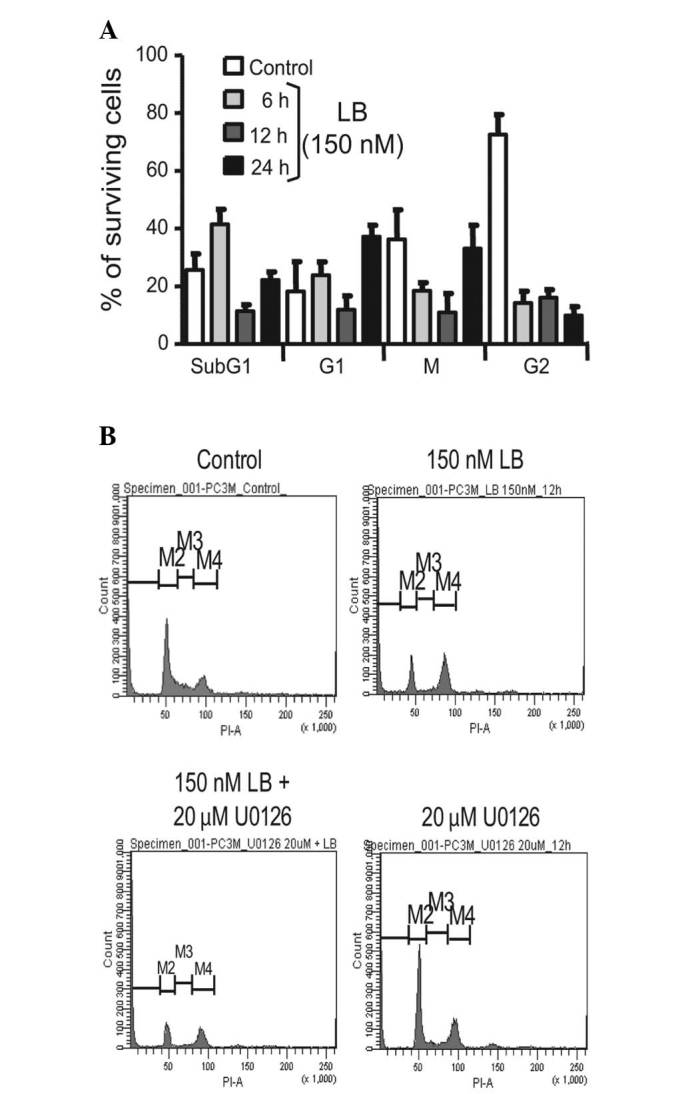
Analysis of LB- and/or MEK inhibitior-treated PTEN-null PC3M cell cycle by flow cytometry. (A) Arrest of cell cycle progression. The cell cycle of the PC3M cells treated with 150 nM LB for 6, 12 and 24 h was analyzed by flow cytometry. (B) Effects of LB and MEK inhibitior on cell cycle progression. Flow cytometry was performed to analyze the cell cycle of PC3M cells treated with LB (150 nM) and/or U0126 (20 μM). The control indicates cells subjected to the vehicle treatment. The DNA was stained with PI and the content was determined by flow cytometry, as described in Materials and methods. LB, latrunculin B; MEK, mitogen-activated protein kinase kinase; PTEN, phosphatase and tensin homolog; PI, propidium iodide.

To analyze cell cycle progression in the PC3M cells treated with 150 nM LB, measurement of the DNA content was performed using flow cytometry. The results showed that LB arrested the cell cycle at the G2 phase compared with that of the control (Fig. 5A). However, addition of U0126 to the LB-treated cells did not affect the DNA content compared with that of the controls (Fig. 5B), indicating that inhibition of the MAPK pathway does not influence cell cycle progression.

Therefore, the results suggested that counteracting the activation of pAkt by MEK/Erk inhibition reduces apoptotic events through actin disruption and not through the PI3K signaling pathway and ROS production in PC3M cells.

## Discussion

Uncontrolled growth of cancer cells is associated with modification of actin dynamics and proteins involved in the regulation of actin polymerization. Thus, actin dynamics have been regarded as a principal regulator of apoptosis and as a target for anticancer chemotherapy (15,16). Actin-disrupting agents, such as LB, jasplakinolide and pectenotoxin, have been used to induce apoptosis in the study of the molecular mechanism of actin disruption and apoptosis in human cancer cells (15,16). Upon treatment with LB, the PC3M cells in the present study showed prominent features of apoptosis, including cytoplasmic shrinkage, marked convolution of the cellular surface, actin disruption, fragmentation of nuclear chromatin and cleavage of PARP, the main substrate of caspase. These effects were shown to be mediated through disruption of the actin cytoskeleton and damage to the DNA, with DSBs being the most destructive, leading to genomic instability, which is detrimental to the cell, inducing the mitochondrial pathway of apoptosis (17). Genotoxic signals are known to activate Bax protein release of cytochrome *c* from mitochondria, resulting in inactivation and subsequent cleavage of PARP (18). As soon as a DSB is generated, the PI3K-related kinases phosphorylate histone H2AX to form γH2AX at the nascent DSB sites (19). Recruitment of γH2AX serves as the binding sites for checkpoint and repair proteins, and influences the chromatin structure that facilitates DNA repair events (20). These results support the induction of apoptosis by actin dysfunction, through the formation of DSBs in PC3M cells.

For cells with DNA damage, cell cycle arrest is important for rendering time for repair of lesions. This study identified that cell cycle arrest at the G2 phase occurred in the PC3M cells treated with LB. This appeared to be the result of LB-mediated DSBs, leading to recruitment of γH2AX around the DSB regions and stabilization of p53 via an ATM-dependent pathway, which in turn resulted in cell cycle arrest. However, minor changes in the level of p21^WAF1/CIF1^ upon treatment with LB (data not shown) suggested that it was not involved in G2 arrest. Alternatively, it may implicate signaling through GADD45 or 14-3-3σ, other downstream signaling molecules of p53. The findings of this study are novel in that phosphorylation of the histone H2AX occurred in cells arrested at the G2 boundary, in contrast to the role of the actin cytoskeleton undergoing early mitosis along with microtubules in eukaryotic cells.

The PI3K/PTEN/Akt and Ras/MEK/Erk signaling pathways have critical roles in human cancer cells (21). Inhibition of the signaling pathways is important for cell survival and induction of apoptosis in cancer cells. Erk1/2, a member of the MAPK kinase family, regulates the cell cycle and progression of apoptosis (22). The effect of treatment with LB observed in the PC3M cells was consistent with previous results showing activation of Erk and cell cycle arrest at the G2 stage (15,23). LB-induced cell cycle arrest at the G2 phase was also observed in yeast and mammalian cells (15,24,25), suggesting the involvement of Erk1/2 activation in regulation of actin disruption for mitosis onset control. However, treatment of cells with DNA-damaging reagents, such as hydroxyl urea, resulted in induced phosphorylation of Erk, although the cell cycle was arrested at S phase (2). To study the detailed aspects of actin disruption-mediated apoptosis signaling, cells were treated with NAC, an antioxidant, and U0126, a highly selective inhibitor of the MEK/ERK pathway (26). While PC3M cells treated with LB alone showed typical features of apoptosis, including cytoplasmic shrinkage, cells treated with both LB and NAC did not show any morphological changes associated with apoptosis. By contrast, cells treated with LB and U0126 showed markedly suppressed apoptotic features. Similar results were obtained for PARP cleavage, as indicated by its induction in the cells treated with LB and U0126, but not in cells treated with LB and NAC. The results indicated that LB-mediated apoptosis includes induction of DSBs via the MEK/Erk pathway, with little effect of Erk1/2 inhibition on G2 phase cell cycle arrest in PC3M cells.

Additionally, inhibition of RAS/MAPK signaling by PD325901, an MEK inhibitor, resulted in significantly reduced metastatic progression initiated from transplanted stem or progenitor cells (27) ), similar to our results showing an association between actin and Erk signaling. The elevated expression of Akt due to PTEN gene deletion resulted in suppression of the Raf/MEK/Erk pathway, leading to terminal differentiation of PC3M cells (28). A markedly reduced level of pAkt expression was observed upon co-treatment with LB and U0126, compared with that of LB treatment alone. Previous results have indicated that treatment with LB leads to apoptosis through ROS-mediated signaling in MCF-7 cells (15). The current results are unique in that they demonstrate actin disruption-mediated Erk activation and counteracting activation of pAkt by MEK/Erk inhibition in PC3M cells. Collectively, activation of RAS/MAPK signaling serves as a potentiating second hit to alteration of the PTEN/PI3K/AKT axis, and co-targeting both pathways is highly effective in preventing development of metastatic prostate cancers.

Therefore, the findings indicate that activation of RAS/MAPK signaling serves as a potentiating second hit for alteration of the PTEN/PI3K/AKT axis, and co-targeting both pathways is highly effective in preventing development of metastatic prostate cancers. These findings will not only broaden knowledge of the tumorigenesis mechanism in various malignant types of cancer, but also aid the development of an anticancer chemotherapy targeting actin disruption in highly metastatic cancer.
